# The Hidden Factor—Low Quality of Data is a Major Peril in the Identification of Risk Factors for COVID-19 Deaths: A Comment on Nogueira, P.J., et al. “The Role of Health Preconditions on COVID-19 Deaths in Portugal: Evidence from Surveillance Data of the First 20293 Infection Cases”. *J. Clin. Med.* 2020, *9*, 2368 

**DOI:** 10.3390/jcm9113442

**Published:** 2020-10-27

**Authors:** Cristina Costa-Santos, Inês Ribeiro-Vaz, Matilde Monteiro-Soares

**Affiliations:** 1 Department of Community Medicine, Information and Health Decision Sciences–MEDCIDS, Faculty of Medicine, University of Porto, 4200-450 Porto, Portugal; inesribeirovaz@gmail.com (I.R.-V.); mat.monteirosoares@gmail.com (M.M.-S.); 2 Center for Health Technology and Services Research–CINTESIS, Faculty of Medicine, University of Porto, 4200-450 Porto, Portugal; 3 Porto Pharmacovigilance Centre, Faculty of Medicine, University of Porto, 4200-450 Porto, Portugal

We read with great interest the article by Nogueira P.J. and colleagues [1] about the identification of factors associated with COVID-19 deaths in Portugal.

The influence of age in increasing the chances of mortality from COVID-19 is expected, but the authors reported very high odds ratios (OR)—50.9, 70.7, 83.2, 91.8 and even 140.2—for the different age groups, with age under 56 years old as the reference. On the other hand, comorbidities, such as cardiac disease and kidney and neuromuscular disorders, had much less weight in increasing the chances of death, with ORs of 2.86, 2.85 and 1.58, respectively. Moreover, in this model, diabetes was not significantly associated with COVID-19 mortality, which is unexpected [2,3,4]. We believe that these extremely high numbers for age and the low effects of some comorbidities are probably related to the poor quality of the dataset used.

The authors developed their models by analyzing an official dataset provided by the General Health Directorate of Portugal (DGS) made available to research groups on the 27 April 2020. This dataset was made available by the DGS upon request and required the submission of a project protocol and an ethical committee approval [5]. 

This dataset has important data quality problems. We had requested and gained access to the dataset to answer another research question, but when we started the assessment of the data quality, it became evident that there were problems with the data. We will use an example to support our views. Recently, an update of this database, presenting the same cases plus those who tested positive for COVID-19 in May and June and correcting some information, was sent by the DGS to the research groups who had requested the first dataset. In the updated version of the dataset, the proportion of people with no comorbidities changed massively, even in the data that were already available in the first version. Globally, in the first version of the dataset, 83% of COVID-19 cases were recorded as having “no comorbidities” and there were no cases with missing information about whether or not the patient had comorbidities, while in the updated version of the dataset, only 32% of the cases were recorded as having “no comorbidities” and 46% of the cases had missing information about whether or not the patient had comorbidities.

This implies that in the first version of the dataset (used by Nogueira P.J. and colleagues [1]), COVID-19 cases for which there was no information about comorbidities were recorded as not having any comorbidity, and this may explain the unexpectedly high OR for the age groups reported and lack of (or low) impact of comorbidities on the odds of dying. Of the COVID-19 cases older than 55 years reported in the first database as having no comorbidities, about 41% have, in fact, no information about comorbidities; those cases may have comorbidities, and we cannot assume that they have no comorbidities. Of the cases analyzed by Nogueira P.J. and colleagues recorded as without comorbidities, 41% are missing cases and should not have been included in their final model.

Even though the dataset had other quality-of-data problems, we describe the errors related to incomplete information regarding comorbidities as an example to highlight the perils of modelling risk factors using low-quality datasets. This example shows the need for data curation procedures by trained data scientists. The urgency of producing evidence that may help to tackle COVID-19 requires broad collaboration to produce high-quality datasets to be used in statistical modelling; otherwise, the reliability of results is limited by an unapparent or hidden factor—data quality. 
